# Prognostic value of right ventricular diastolic dysfunction in patients with inferior ST-elevated myocardial infarction

**DOI:** 10.1186/s43044-023-00350-9

**Published:** 2023-04-21

**Authors:** Ahmed Mahmoud El Amrawy, Shaimaa Abd ElKhalek Zaghloul, Eman Mohamed El Sharkawy, Mohamed Ahmed Sobhy

**Affiliations:** Cardiology Department, Faculty of Medicine, Alexandria University, Medical Campus, Champlion Street, El-Azarita, Alexandria, Egypt

**Keywords:** ST elevation myocardial infarction, Right ventricle infarction, Diastolic dysfunction, Tissue Doppler imaging

## Abstract

**Background:**

Right ventricle infarction (RVI) is predominantly a complication of inferior wall myocardial infarction; it occurs in approximately one third of these patients. Right ventricular dysfunction in patients with inferior STEMI and RV infarction was under assessed. Nevertheless, studies which targeted RV assessment by echocardiography, did not routinely evaluate RV diastolic dysfunction. In this study, we aimed to evaluate RV diastolic dysfunction and its prognostic value in patients with inferior STEMI and RVI.

**Results:**

Sixty patients with inferior STEMI and RV infarction, who underwent primary PCI were enrolled in the study. Patients with pre-existing clinical conditions that might affect RV function, were excluded. Echocardiography was performed within twenty-four hours following the PCI, to assess the RV systolic and diastolic functions with special focus on tricuspid inflow velocities (E velocity, A velocity and E/A ratio) by pulsed wave (PW) doppler and tricuspid annular velocities by tissue doppler index (TDI) (E′, A′ and E/E′ ratio). Clinical features and MACE, including cardiogenic shock, arrhythmia, stroke, reinfarction and death were analyzed in all our patients within 3 months follow up period. The average age of the study population was 51.58 ± 10.11 years, 10% were females. Five patients developed MACE (death, cardiogenic shock and pulmonary edema, anterior STEMI and cardiogenic shock, recurrent inferior STEMI, and arrhythmia and stroke), of whom four occurred in hospital within the first 48 h. Patients who developed MACE had high filling pressures, as all of them had E/E′ > 6. E′ velocity ≤ 6 cm/sec was associated with increased MACE as 25% of patients with E′ velocity ≤ 6 had MACE compared with 2.3% of patients with E′ velocity > 6 with a *p* value of 0.015.

**Conclusions:**

Tricuspid annular velocities by TDI are essential when evaluating RV diastolic dysfunction. E/E′ and E′ velocity have a prognostic value in patients with inferior STEMI and RV infarction; E/E′ > 6 and E′ velocity ≤ 6 cm/sec were associated more MACE in patients with inferior STEMI and RVI.

## Background

Patients with inferior ST-elevation myocardial infarction (STEMI) have more favorable prognosis than patients with anterior STEMI, although certain associations may indicate a worse outcomes and increased mortality. High risk groups of inferior STEMI patients are those with heart block, right ventricle (RV) infarction, cardiogenic shock and cardiac arrest due to ventricular tachycardia or ventricular fibrillation. Right ventricle infarction is predominantly a complication of inferior wall myocardial infarction (IWMI); it occurs in approximately one third of these patients. In about one half of patients with RV infarction, it is of hemodynamic significance. RV infarction is associated with anterior myocardial infarction in less than 10% of cases; isolated RV infarction is rarely seen [1, 2]. Patients with RV infarction may develop the typical triad of hypotension, clear lung fields, and increased jugular venous pressure [3]. As a result of right ventricular infarction, the RV compliance decreases, RV filling is reduced and so the RV stroke volume. RVI may be masked by left ventricular systolic dysfunction with hypotension and pulmonary congestion [4]

There is still little information regarding RV diastolic function & numerous gaps remain in our understanding. In ischemic patients, the pathophysiologic response is a failure of the early rapid filling to increase and a heightened dependency on atrial contribution and consequent elevated right atrial (RA) pressure, marked diastolic dysfunction with elevated filling pressures occurs in case of acute RV myocardial infarction [5–7].

Marked diastolic dysfunction with elevated filling pressures occurs in case of acute RV myocardial infarction. RV diastolic dysfunction serves as an early and more easily quantifiable marker of subclinical RV dysfunction. Multiple studies have shown that RV diastolic dysfunction is usually present before apparent systolic dysfunction and before RV dilatation or RV hypertrophy (RVH). Tricuspid annular velocities are higher than the mitral annular velocities. E/E′ ratio can be used to estimate RV filling pressures & diastolic function, RV E/E′ ratio greater than 6 suggests a RA pressure greater than 10 mmHg [5, 8–10].

### Aim of the study

Evaluation of RV diastolic function and its prognostic value in patients with RV infarction in the setting of inferior STEMI.

## Methods

### Patient population

Patients, who were admitted with Inferior STEMI and RV Infarction to two tertiary hospitals (University Hospitals) between October 2019 and December 2020, were enrolled in this prospective study. The diagnosis was established on clinical basis, enzyme changes, 12-lead electrocardiogram (ECG) and Echocardiography. The inclusion criteria were: (a) Onset of symptoms < 24 h before Primary PCI; (b) ST-segment elevation > 0.1 mV in two contiguous inferior leads, II, III, or aVF, and ST-segment elevation > 0.1 mV in V3R or V4R.; (c) Right coronary artery occlusion proximal to the major right ventricular (RV) branches at base line coronary angiography; (d) All patients who were treated with primary PCI. Exclusion criteria were: (1) Patients with concurrent pericardial disease; (2) Atrial fibrillation on admission; (3) Previous history of RV dysfunction; (4) Previous history of chronic pulmonary disease, pulmonary hypertension or acute pulmonary embolism; and valvular heart disease (moderate or severe insufficiency and/or stenosis).

Following admission, all patients were subjected to full history taking, clinical examination, blood sampling including cardiac enzymes, ECG, PPCI and echocardiography.

RV infarction was diagnosed clinically by hypotension (defined as systolic blood pressure below 90 mmHg lasting more than 15 min), clear lungs, and elevated Jugular venous pressure.

### Coronary angiography and PPCI

All patients received dual antiplatelets (aspirin and ticagrelor or clopidogrel, according to which P2Y12 inhibitor is available in the emergency room at the time of presentation), and parenteral anticoagulants and PPCI was performed in accordance with ESC guidelines of myocardial revascularization [1]. Coronary angiography revealed proximal right coronary artery (RCA) occlusion (osteal, para-osteal or proximal). PPCI success was determined using angiographic; reduction of minimum stenosis diameter, assessed by angiography, to less than 20% with TIMI III flow, procedural; absence of major clinical complication (e.g., re-infarction and emergency CABG) which necessitate target vessel revascularization during the hospital stay and clinical parameters; relief of signs and symptoms of ischemia.

### Echocardiographic evaluation

Echocardiography study was performed with the patient either supine and/or in the left lateral decubitus position, using a digital ultrasonic device system (SIEMENS ACUSON X700) within 24 h after the PPCI.

We analyzed left ventricle ejection fraction (LVEF) (by 2D biplane method), RV FAC, TAPSE, PW doppler velocities (E vel, A vel, DT and E/A), TDI velocities (E′, A′, E/E′), right atrial (RA) area, IVC diameter and collapsibility and pulmonary artery systolic pressure (PASP) in all patients [5].

Three months clinical follow-up was obtained and occurrence of ventricular arrhythmia and major adverse cardiovascular events [MACE] (death, cardiogenic shock, MI and stroke) was recorded.

We divided our studied patients to two groups: MACE patients and non-MACE patients; we compared between these groups according to demographic data, risk factors and echocardiographic parameters.

### Statistical analysis of the data

Data were fed to the computer and analyzed using IBM SPSS software package version 20.0. (Armonk, NY: IBM Corp). The Kolmogorov–Smirnov was used to verify the normality of distribution of variables. Comparisons between groups for categorical variables were assessed using Chi-square test, Fisher’s Exact or Monte Carlo correction were used as a correction for chi-square when more than 20% of the cells have expected count less than five. Student's *t*-test was used to compare two groups for normally distributed quantitative variables, while Mann–Whitney test was used to compare between two groups for abnormally distributed quantitative variables. Positive correlation tests using ROC curve and AUC were used to estimate the cut off value for E′ velocity that is correlated to MACE. *p* value less than 0.05 was considered significant.

## Results

The study included 60 patients, of whom 6 patients (10%) were females. The average age of the study population was 51.58 ± 10.11 years. Regarding significant demographic data, adverse events were more common in females. Five patients out of the enrolled 60 patients developed MACE (death, cardiogenic shock and pulmonary edema, anterior STEMI and cardiogenic shock, recurrent inferior STEMI, and arrhythmia (AF) and stroke). Ninety percentage of MACE developed in the hospital in the 48 h following the admission. Forty percentage of MACE group were females compared to 7.3% in the non-MACE group with a significant *p*-value of 0.020 (Table 1).

**Table 1:** Comparison between MACE and non-MACE groups according to different parameters and risk factors

	Total (*n* = 60)	Follow up for MACE		Test of sig.	*p*
		Absent (*n* = 55)	Present (*n* = 5)		
Sex					
Female	6 (10%)	4 (7.3%)	2 (40.0%)	*χ*^2^ = 5.455^*^	0.020^*^
Male	54 (90%)	51 (92.7%)	3 (60.0%)		
Age (years)					
Mean ± SD	51.6 ± 10.1	51 ± 10.1	58.4 ± 8.4	*t* = 1.596	0.116
Median (Min.–Max)	53.5 (26–70)	51 (26–70)	60 (47–70)		
Diabetes mellitus	20 (33.3%)	15 (27.3%)	5 (100.0%)	*χ*^2^ = 10.909^*^	0.001^*^
Hypertension	20 (33.3%)	17 (30.9%)	3 (60.0%)	*χ*^2^ = 1.745	0.186
CKD	9 (15.0%)	6 (10.9%)	3 (60.0%)	*χ*^2^ = 8.663^*^	0.003^*^
Dyslipidemia	23 (38.3%)	19 (34.5%)	4 (80.0%)	*χ*^2^ = 4.006	0.045^*^
Smoking (current)	51 (85.0%)	49 (89.1%)	2 (40.0%)	*χ*^2^ = 8.663^*^	0.003^*^
Smoking (former)	2 (3.3%)	2 (3.6%)	0 (0.0%)	*χ*^2^ = 0.188	0.665
Alcohol	2 (3.3%)	2 (3.6%)	0 (0.0%)	*χ*^2^ = 0.188	0.665
Family history of CAD	18 (30.0%)	18 (32.7%)	0 (0.0%)	*χ*^2^ = 2.338	0.126
Hemoglobin level (g/dl)					
Mean ± SD	13.5 ± 2.1	13.7 ± 2	11.5 ± 1.6	*t* = 2.412^*^	0.019^*^
Median (Min.–Max)	14 (8.5–16)	14 (8.5–16)	12 (9–13.4)		
S. Creatinine (mg/dl)					
Mean ± SD	1 ± 0.8	1 ± 0.9	1 ± 0.1	U = 62.0^*^	0.042^*^
Median (Min.–Max)	0.9 (0.5–7)	0.9 (0.5–7)	1.1 (0.9–1.2)		
Positive Troponin	60 (100%)	55 (100%)	5 (100%)	–	–
LDL level (mg/dl)					
Mean ± SD	118 ± 24.1	115.8 ± 23.2	142.8 ± 20.4	*t* = 2.511^*^	0.015^*^
Median (Min.–Max)	109.5 (86–177)	107 (86–177)	143 (111–165)		

*SD*, Standard deviation; *t*, Student *t*-test; *U*, Mann Whitney test; *χ*^2^, Chi square test; *p*, *p* value for comparing between the studied groups

*Statistically significant at *p* ≤ 0.05

Regarding risk factors, 85% of all patients were current smokers; smoking was apparently less in MACE patients (*p* = 0.003), 40% compared to 89.1% in non-MACE patients. Hundred percentage of MACE group had diabetes (DM) (*p* = 0.001), 60% had chronic kidney disease (CKD) (*p* = 0.003), and 80% had dyslipidemia (*p* = 0.045). Also, LDL was significantly higher in patients who had MACE (*p* = 0.015) (Table 1).

The ECG showed ST elevation in leads II, III, and aVF ≥ 1.0 mm with reciprocal ST depression in I and aVL and ST-segment elevation ≥ 1.0 mm in lead V4R alone or in both V3R and V4R in all patients, the elevation was transient in 40% of the patients (24 patients), only 10 patients showed 0.1 mV ST segment elevation in V1, and 5 patients showed ST segment elevation in posterior leads.

PCI procedure: All patients (100%) received oral dual antiplatelets (DAPT) before the procedure. Forty patients (66.66%) received ticagrelor and the other 20 patients (33.34%) received clopidogrel. Regarding the pain to balloon time: primary PCI was performed within 24 h from onset of chest pain, and the culprit lesion was proximal RCA occlusion (osteal, para-osteal or proximal) in all patients.

Echocardiography (Table 2): Regarding the LV systolic function, LVEF was significantly lower in MACE group as it ranged from 30 to 48% with a mean of 38.40 ± 7.37% compared to non-MACE group ranging from to 25–58% with a mean of 46.09 ± 7.24% with a *p* value 0.027. As regards the RV systolic function, it was significantly lower in MACE group as fractional area change (FAC) ranged from 23 to 40% with a mean of 29.0 ± 7.31% compared to non-MACE group ranging from 20 to 50% with a mean of 37.91 ± 6.97% (*p* = 0.008). IVC diameter and pulmonary artery systolic pressure (PASP) were higher in MACE group, but not statistically significant.

**Table 2:** Comparison between MACE and non-MACE groups regarding echocardiographic parameters

	Total (*n* = 60)	Follow up for MACE		Test of sig.	*p*
		Absent (*n* = 55)	Present (*n* = 5)		
LVEF (%)					
Mean ± SD	45.5 ± 7.5	46.1 ± 7.2	38.4 ± 7.4	*t* = 2.271*	0.027*
Median (Min.–Max)	45 (25–58)	46 (25–58)	35 (30–48)		
RV					
ESA (cm^2^)					
Mean ± SD	12 ± 3.5	11.7 ± 3.3	15 ± 4.6	*t* = 2.028*	0.047*
Median (Min.–Max)	11 (6–20)	11 (6–20)	15 (8–20)		
EDA (cm^2^)					
Mean ± SD	18.9 ± 3.9	18.7 ± 3.8	21.2 ± 4.8	*t* = 1.386	0.171
Median (Min.–Max)	18 (10–29)	18 (10–29)	20 (15–27)		
FAC (%)					
Mean ± SD	37.2 ± 7.4	37.9 ± 7	29 ± 7.3	*t* = 2.726*	0.008*
Median (Min.–Max)	39.5 (20–50)	40 (20–50)	25 (23–40)		
TAPSE (cm)					
Mean ± SD	1.67 ± 0.27	1.7 ± 0.3	1.6 ± 0.4	*t* = 0.963	0.340
Median (Min.–Max)	1.7(1–2.2)	1.7 (1–2.2)	1.5 (1.2–2.2)		
RV WMA					
Absent	20 (33.3%)	19 (34.5%)	1 (20.0%)	*χ*^2^ = 0.436	^FE^*p* = 0.656
Present	40 (66.7%)	36 (65.5%)	4 (80.0%)		
E Vel (cm/sec)					
Mean ± SD	49.2 ± 11.7	48.8 ± 11.6	54.2 ± 12.4	*t* = 0.991	0.326
Median (Min.–Max)	50 (24–76)	50 (24–76)	52 (40–74)		
DT (ms)					
Mean ± SD	195.9 ± 41.61	196.8 ± 41.3	186 ± 48.7	*t* = 0.552	0.583
Median (Min.–Max)	190 (100–300)	190 (130–300)	203 (100–220)		
A Vel (cm/sec)					
Mean ± SD	42.2 ± 11.4	42.5 ± 11.5	38 ± 10.8	*t* = 0.757	0.452
Median (Min.–Max)	4 2 (22–70)	41.5 (25–70)	42.5 (22–45)		
E/A				*U* = 86.50	0.524
Mean ± SD	1.2 ± 0.47	1.2 ± 0.5	1.4 ± 0.7		
Median (Min.–Max)	1.2 (0.5–2.5)	1.2 (0.5–2.1)	1.2 (0.9–2.5)		
E′ vel (cm/sec)					
Mean ± SD	7.8 ± 2.1	8 ± 2.1	6.1 ± 0.8	*t* = 1.959	0.055
Median (Min.–Max)	7.3 (5–14)	7.5 (5–14)	6 (5.5–7.5)		
A′ vel (cm/sec)					
Mean ± SD	11.2 ± 3	11.3 ± 3	9.8 ± 1.3	*t* = 0.977	0.333
Median (Min.–Max)	1 1(6–20)	11 (6–20)	10 (8–11)		
s′ velocity (cm/sec)					
Mean ± SD	10.8 ± 1.8	10.9 ± 1.8	9.7 ± 1.5	*t* = 1.384	0.172
Median (Min.–Max)	10.8 (5.8–14.6)	11 (5.8–14.6)	9.5 (8–12)		
E/E′					
Mean ± SD	6.6 ± 2	6.4 ± 1.8	8.8 ± 2.7	*t* = 2.782*	0.007*
Median (Min.–Max)	6.5 (3.2–13.4)	6.1 (3.2–12)	8 (6.6–13.4)		
IVC Diameter (cm)					
Mean ± SD	1.8 ± 0.3	1.8 ± 0.3	2 ± 0.1	*t* = 1.203	0.234
Median (Min.–Max)	1.8 (1–2.5)	1.8 (1–2.5)	2 (1.8–2.1)		
IVC Collapsibility index > 50%	58 (96.7%)	54 (98.2%)	4 (80%)	*χ*^2^ = 2.168	0.03*
SPAP (mmHg)					
Mean ± SD	21.3 ± 8	21.2 ± 8.3	22.6 ± 3.9	*t* = 0.378	0.707
Median (Min.–Max)	20 (10–50)	20 (10–50)	25 (16–25)		

*SD*, Standard deviation; *t*, Student *t*-test; *U*, Mann Whitney test; *χ*^2^, Chi square test; *p*, *p* value for comparing between the studied groups

*Statistically significant at *p* ≤ 0.05

For the PW velocities (Table 2): PW velocities were not statistically different between MACE and non-MACE groups.

Regarding the TDI (tissue doppler imaging) velocities (Table 2), E′ velocity ranged from 5.0 (less than normal limits) to 14.0 cm/s with a decreased mean of 7.83 ± 2.08 cm/s, E/E′ was significantly higher in MACE group as it ranged from 6.60 to 13.4 with a mean of 8.82 ± 2.72 compared to 3.20–12.0 with a mean of 6.38 ± 1.80 in non-MACE group (*p* = 0.007). A′ and S′ velocities were not statistically significant between all groups.

We analyzed the relation between E′ wave velocity with the follow up for MACE, and we found that diastolic dysfunction affected myocardial relaxation velocities significantly as TDI revealed that E′ wave velocity less than or equal to 6 cm/s was associated with more adverse events. We divided our patients into two groups according to E′ wave velocity; one group had E′ ≤ 6 and the other had E′ > 6 cm/s. E′ velocity ≤ 6 cm/s was associated with increased MACE as 25.0% of patients with E′ velocity ≤ 6 had MACE compared with 2.3% of patients with E′ velocity > 6 m/s (*p* = 0.015) (Table 3).

**Table 3:** Relation between E′ vel and E/E′ with Follow up for MACE (*n* = 60)

	E′ vel		E/E′	
	≤ 6 (*n* = 16)	> 6 (*n* = 44)	≤ 6 (*n* = 25)	> 6 (*n* = 35)
Follow up for MACE				
Absent	12 (75%)	43 (97.7%)	25 (100%)	30 (85.7%)
Present	4 (25%)	1 (2.3%)	0 (0%)	5 (14.3%)
*χ*^2^ P value	7.934* (0.015*)		3.896* (0.048*)	

*χ*^2^, Chi square test; *p*, *p* value for comparing between the studied groups

*Statistically significant at *p* ≤ 0.05

We also analyzed the relation between E and E′ and the occurrence of MACE (Table 3), we classified our studied cases into two groups; one group with E/E′ ≤ 6 (25 patients) and the other with E/E′ > 6 (35 patients); E/E′ > 6 was associated with increased MACE and all patients who developed MACE had E/E′ > 6 which was statistically significant (*p* = 0.048).

## Discussion

When reviewing the literature, many studies are found discussing RV systolic function and fewer discussing the RV diastolic function. RV diastolic function interpretation is very difficult and challenging. RV diastolic function is affected by respiration, circulating volume in intravascular spaces, LV function and filling pressures, preexisting pulmonary hypertension, and ventricular interdependence [8, 9]. RV diastolic dysfunction is defined by increased right ventricular filling pressures, caused by passive (RV chamber stiffness) and active (impaired RV relaxation) dysfunction of ventricular muscle function during diastole [7, 9, 10].

The parameters used to assess RV diastolic function are essentially the same as those used to assess the left side: Doppler velocities of the Trans tricuspid flow (E, A, and E/A), tissue Doppler velocities of the tricuspid annulus (E′, A′, E′/A′) and deceleration time. The tricuspid E/E′ ratio is highly recommended to accurately assess RV diastolic function [11] Trans-tricuspid Flow (TTF) velocities are characterized by that they tend to be lower than-trans mitral flow velocities owing to the larger TV annular size and they vary during spontaneous respiration by 20%. E/A ratio is increased during inspiration due to an increase in E wave velocity [12]. Tricuspid annular velocities by TDI are higher than the mitral annular velocities and E/E′ ratio can be used to estimate RV filling pressures (E/E′ ratio greater than 6 suggest an RA pressure greater than 10 mmHg) [5, 12]. The severity of the hemodynamic abnormalities, associated with RV infarction, is related to the extent of RV ischemia and consequent RV dysfunction as well as to the restraining effect of the pericardium, LV function, and ventricular interdependence. Also, augmented atrial contraction is necessary to overcome the stiffness of the ischemic RV, and factors that impair RV filling (intravascular volume depletion, concomitant atrial infarction, loss of atrioventricular synchrony) may severely compromise hemodynamics and result in cardiogenic shock [10]

In our study, we included 60 patients with inferior STEMI and RV infarction. All of them were treated by PPCI. Females were less represented (10%). Smoking was the most common risk factor, 51 patients (85%) were smokers, but most reports showed that only 40–45% of STEMI patients were recent smokers. Despite this increased prevalence, there was no difference in the outcomes between smokers and nonsmokers. The studied population has an increased prevalence of smoking in comparison to other populations [13]. All of our cases who developed MACE (5 patients), had DM. This may reflect the burden of DM, especially the uncontrolled DM, and its danger being a common risk factor of ischemic heart diseases. DM was associated with worse outcomes in all previous studies of STEMI [14]. Also, LV function was lower in patients with MACE. Antoni et al. and Kakouros et al. showed that patients with RV involvement in inferior AMI were at increased risk of adverse events [15, 16].

According to ASE guidelines for assessment of right heart in adults, the tricuspid E/E′ ratio has been shown to correlate well with hemodynamic parameters. We used tissue doppler and other parameters, e.g., IVC collapsibility to identify patients with high filling pressures [5, 12]. Thirty five patients had E/E′ > 6 and five of them had non collapsible IVC. High filling pressures are associated with moderate or severe diastolic dysfunction. We classified our cases into two groups, the first included patients with E/E′ > 6 and the other included patients with E/E′ ≤ 6 then we compared between both according to presence or absence of MACE. All of our 5 patients who developed MACE had E/E′ > 6 with a significant *p* value of 0.048.

Restrictive pattern of RV diastolic function by PW doppler (E/A ratio > 2 and DT < 120), was absent in our cases. The single case showed this pattern was excluded due to frequent PVCs (bigeminy). No cases in our study showed this pattern even in the presence of RV systolic dysfunction or LV diastolic dysfunction grade 3 (LV restrictive pattern does not necessitate severe RV diastolic dysfunction). By reviewing the literature, most of studies diagnosed RV severe diastolic dysfunction (DD) by right heart catheterization. They did not rely on PW doppler for DD diagnosis. Mertens et al. studied indices of right ventricular (RV) diastolic function in patients with tetralogy of Fallot (TOF). The purpose of this study was to determine echocardiographic predictors of severe RV diastolic dysfunction by comparing the following indices (tricuspid E/A, E/E′, deceleration time, pulmonary artery forward flow, dilated inferior vena cava (IVC), and hepatic vein diastolic flow reversal (HVDFR) with the results of right heart catheterization. Of the indices assessed, dilated IVC had the best sensitivity of 95% while HVDFR had the best specificity of 69% for detecting severe RV diastolic dysfunction [17]. Also, Konstam et al. [10] found that RV diastolic dysfunction may be measured with early diastolic tricuspid inflow (E) and tissue Doppler early diastolic myocardial velocity at the tricuspid lateral annulus (E′) ratio (E/E′). So, by reviewing the literature, E/A ratio for evaluation of RV diastolic function is neither conclusive nor reliable [10, 11].

As E/A ratio, E velocity and A velocity by pulsed wave doppler on right side vary significantly with respiration and fluid status, tissue doppler is a better alternative. The less the E′ velocity, the higher the incidence of MACE. E′ may be the only indicator of severe RV diastolic dysfunction. What is the cutoff point? In our study it was 6 cm/s. We classified the patients into two groups, first group E′ is less than or equal to 6 (≤ 6) and the other E′ > 6. The group with E′ ≤ 6 was associated with increased MACE in comparison to the group with E′ > 6 with a statistically significant *p* value 0.015. Screening of patients by E′ may be useful in anticipating MACE.

MACE developed in 5 patients of which 4 patients complicated during hospital stay. B Altıntaş also reported that all the MACE in his study developed during the hospital stay [18]

We performed our echocardiography study within 24 h following the PPCI. We considered it unethical to delay the PCI of patients with myocardial infarction to perform a complete echocardiography study before, so we do not know the RV diastolic function at the time of admission or before successful reperfusion.

In our study, one patient died of arrhythmia (AF) and stoke. By reviewing the past articles of IWMI (inferior wall myocardial infarction), RV infarction was associated with increased arrhythmia in comparison to cases of IWMI without RV infarction [19]. Female patients were 6 out of 60 patients, 2 of them developed MACE. This is a very high ratio in females compared to male patients as 33.3% of female patients developed MACE versus 3.7% in male patients. Complication rates after MI are higher in women than in men despite similar success rates with treatment [20].

## Conclusions

There is still little information regarding RV diastolic dysfunction (DD) and its grading in patients with RVI. Marked diastolic dysfunction with elevated filling pressures occurs in case of acute RV myocardial infarction. RV DD serves as an easily quantifiable marker of RV dysfunction and can be used for its prognostic significance in patients with inferior STEMI and RV infarction. Tissue Doppler in the evaluation of RV DD is inevitable. In our study, E′ < 6 cm/sec and E/E′> 6 were associated with increased MACE. E′ and E/E′ should be routinely used during the evaluation of the right ventricle in cases of RV infarction.

### Study limitations

- Small number of included patients.
- Strain imaging was not performed in the included patients.
- No MRI or right heart catheterization to verify or compare our results.

## Data Availability

The datasets used and/or analyzed during the current study are available from the corresponding author on reasonable request.

## Abbreviations

ACS: Acute coronary syndrome
AF: Atrial fibrillation
AUC: Area under the curve
BMI: Body mass index
Bpm: Beats per minute
DAPT: Dual antiplatelets therapy
DD: Diastolic dysfunction
DM: Diabetes mellitus
ECG: Electrocardiography
FAC: Fractional area change
HTN: Hypertension
IVC: Inferior vena cava
IWMI: Inferior wall myocardial infarction
MACE: Major adverse cardiac events
MI: Myocardial infarction
MRI: Magnetic resonance imaging
PA: Pulmonary artery
PASP: Pulmonary artery systolic pressure
PCI: Percutaneous coronary intervention
RA: Right atrium
RIMP: Right ventricular index of myocardial performance
ROC: Receiver operating characteristic curve
RV: Right ventricle
RVDD: Right ventricle diastolic dysfunction
RV EDA: RV end diastolic area
RV ESA: RV end systolic area
RVEF: Right ventricular ejection fraction
RVI: Right ventricular infarction
RVMI: Right ventricular myocardial infarction
RVOT: Right ventricular outflow tract
RWMAs: Regional wall motion abnormalities
STEMI: ST segment elevation myocardial infarction
TAPSE: Tricuspid annular plane systolic excursion
TDI: Tissue Doppler imaging
TIMI: Thrombolysis in myocardial infarction
UFH: Unfractionated heparin
